# Recent Progress of Novel Organic Near‐Infrared‐Emitting Materials

**DOI:** 10.1002/smsc.202200029

**Published:** 2022-07-13

**Authors:** Ming-Peng Zhuo, Xue-Dong Wang, Liang-Sheng Liao

**Affiliations:** ^1^ Institute of Functional Nano & Soft Materials (FUNSOM) Jiangsu Key Laboratory for Carbon-Based Functional Materials & Devices Soochow University Suzhou 215123 China; ^2^ College of Textile and Clothing Engineering Soochow University Suzhou 215123 China; ^3^ Macao Institute of Materials Science and Engineering Macau University of Science and Technology Taipa 999078 Macau SAR China

**Keywords:** charge transfer interactions, energy gap law, near-infrared emission, organic cocrystals, organic semiconductors

## Abstract

Organic near‐infrared (NIR) emissive materials integrating the intrinsic NIR light advantages and organic semiconductor characters have generated immense scientific interest in fundamental science and practical application, including optical communications, night vision, surveillance, and biomedical applications. Nevertheless, the intrinsic energy gap law engenders a huge challenge to simultaneously meet longer emission wavelength and higher luminescence efficiency for organic NIR emitters. Herein, it is suggested that the purposefully molecular design and crystal engineering for novel organic NIR materials are urgently studied and explored. Herein, recent advances in novel organic NIR materials, mainly focusing on the hybridized local and charge transfer (HLCT) compounds, thermally activated delayed fluorescence (TADF) emitters, CT cocrystals, neutral π‐radicals, and excited‐state intramolecular proton transfer (ESIPT)‐active materials, are summarized. It is hoped that this perspective can afford a new insight into the fine design and synthesis of the desired organic NIR emitters.

## Graphical abstract

Organic near‐infrared (NIR) materials with high penetration ability and low phototoxicity have attracted considerable research attention for their fundamental science and practical applications. This perspective summarizes the recent advances in rational design and synthesis of the desired organic NIR emitter and gives an outlook, providing inspiration for their rational design and promising applications.

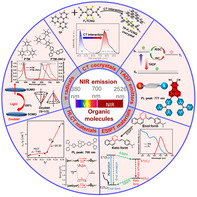

## 1 Introduction

Organic near‐infrared (NIR) emitters with high penetration ability and low phototoxicity have attracted considerable research attention for their fundamental science and practical applications, such as data storage,^[^ ^1^ ^]^ night vision,^[^ ^2^ ^]^ biomedical imaging,^[^ ^3^ ^]^ remote sensing,^[^ ^4^ ^]^ and optical communication/diagnosis.^[^ ^5^ ^]^ Recently, impressive progress has been made in the organic NIR emitters with an extended emission wavelength or an enhanced photoluminescence quantum yield (PLQY) via the design philosophy and the synthesis methods.^[^ ^6^ ^]^ Representative cases include conjugated polymers,^[^ ^7^ ^]^ organic rare‐earth/noble metal complexes,^[^ ^8^ ^]^ and small‐molecule dyes.^[^ ^9^ ^]^ Considering an advanced work reported by Prof. P.‐T. Chou and co‐workers, a new class of phosphorescent platinum(ii) complexes demonstrated charming NIR emission at 866‐960 nm with a PLQY of 5–12% in solid films.^[2b]^ Nevertheless, the expensiveness and scarcity of these noble metals such as platinum and iridium are intense obstacles for their practical applications.^[^ ^10^ ^]^ Conventional NIR chromophores of conjugated polymers or small‐molecule dyes mostly originated from an extended π‐conjugation structure in the nearly planar macrocyclic aromatic. Due to the tight intermolecular π‐stacking and the strong dipole–dipole interactions, these NIR emitters commonly suffer low‐efficacy emission, which greatly hinders their practical application.^[^ ^11^ ^]^ Therefore, it is imperative to develop the novel organic NIR emitter with high performance.

Significantly, the polar donor–acceptor (D–A) architectures with a powerful charge transfer (CT) interaction afford a promising and operable strategy to narrow the bandgap for desired NIR emission. However, the spatial separations between the highest occupied molecular orbital (HOMO) and lowest unoccupied molecular orbital (LUMO) generally generate the forbidden electronic transition for such a CT state, causing fluorescence quenching. Through introducing local excited (LE) in the CT state, the formed hybridized local and CT (HLCT) excited state demonstrates a big transition dipole moment, as well as a considerable orbit overlap between electron and hole moieties, benefiting for the effective NIR emission.^[^ ^12^ ^]^ Owing to rational molecular design toward the adequate separation of spatial distribution for HOMO and LUMO, the thermally activated delayed fluorescence (TADF) could simultaneously harvest both singlet‐state (S_1_) and triplet‐state (T_1_) excitons via the reverse intersystem crossing (RISC) process from the *T*_1_ to the *S*_1_, which demonstrates the great potential to enhance the emission performance.^[^ ^13^ ^]^ Furthermore, the distinct physical/chemical properties of organic CT complexes could be tailored via manipulating their packing modes, which provides a powerful approach for the effective NIR emission.^[^ ^14^ ^]^ Notably, the excited‐state intramolecular proton transfer (ESIPT) process is a promising alternative for a redshift emission wavelength, especially the NIR emission, via forming two tautomers of enol and keto states.^[^ ^15^ ^]^ Owing to the unusual electronic structures for the formation of spin‐allowed doublet, the organic radical molecules present the potential opportunity in NIR‐emissive applications.^[^ ^16^ ^]^ All of these novel organic NIR emitters are necessary to be summarized, which would be helpful for readers to develop the desired organic NIR emitters via a purposefully molecular design. Herein, we will highlight the significant progress in the finely molecular design of the novel organic NIR emitters, including the HLCT compounds, TADF materials, CT cocrystals, neutral π‐radicals, and ESIPT‐active materials. Importantly, the attention lay emphasis on the molecular design mechanisms and the corresponding structure–emission features.

## 2 Novel NIR‐Emissive Organic Molecules

**Figure 1:**
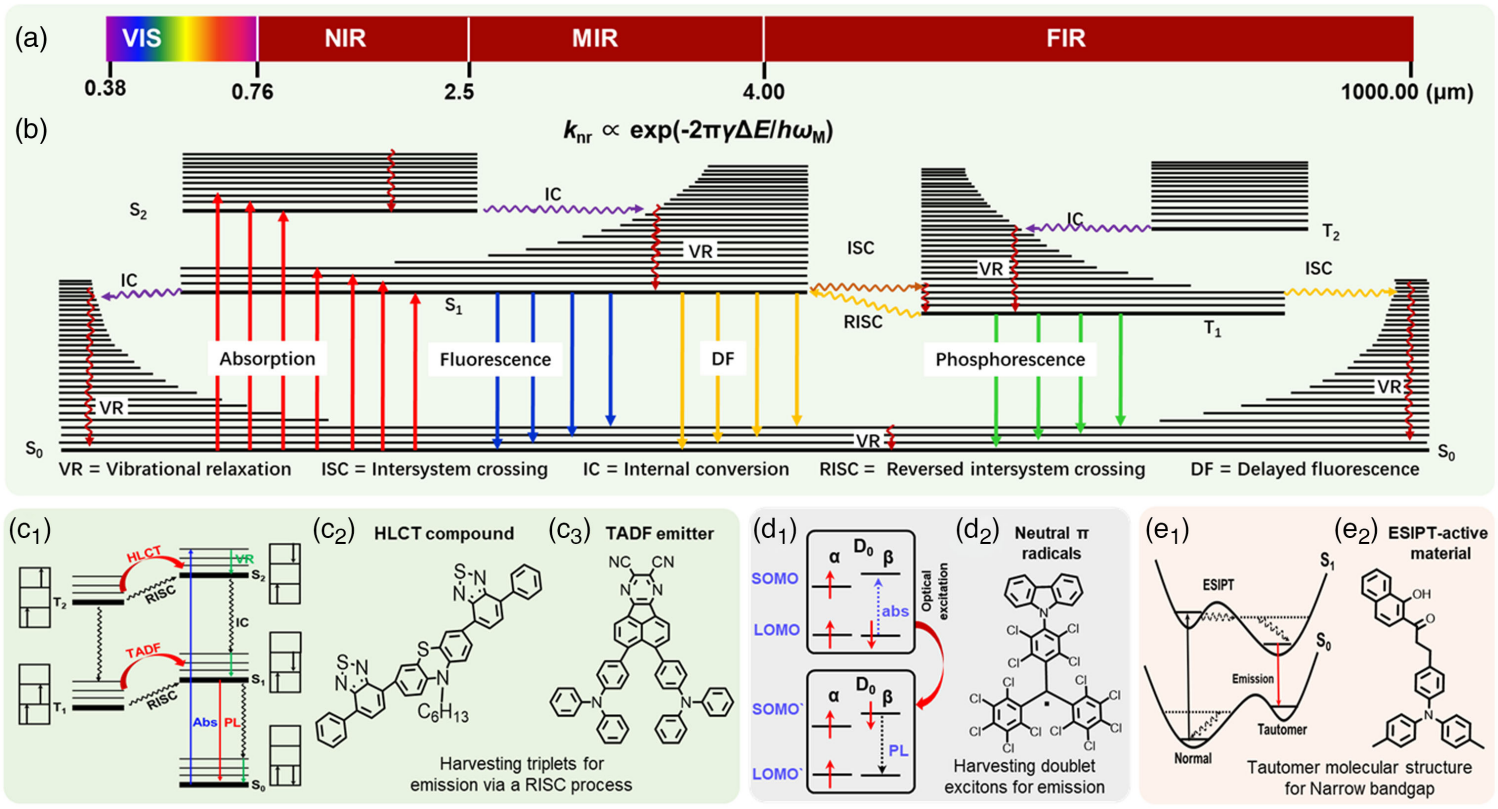
a) Classification of electromagnetic waves of the NIR light. b) Jablonski diagram of the small organic semiconductor molecules. c_1_) Energy diagram of RISC process for HLCT and TADF processes. The molecule structures of c_2_) HLCT compound and c_3_) TADF emitter. d_1_) Doublet emission under photoexcitation and d_2_) the molecule structures of neutral π radical. e_1_) Schematic of a four‐level energy system of ESIPT‐active molecule. e_2_) The molecule structures of ESIPT‐active molecules.

NIR light corresponds to the nominal red edge of the visible spectrum extending to the emission peak ranging from 760 to 2500 nm (**Figure** **1**). Compared with the visible light, the NIR light produces many unique and fascinating preponderances. NIR light possesses a reduced absorption and scattering by skin, blood, and other tissues, which is conductive to deeply penetrating biological tissues with minimum interference and enhanced bioimaging capabilities, rendering NIR emitters particularly appealing for photodynamic therapy, biomedical sensing, and optical imaging.^[^ ^17^ ^]^ Due to the small energy involved with the generation and decay, NIR light demonstrates a limited number of side reactions and a small transmission loss.^[^ ^18^ ^]^ Correspondingly, the NIR emitters are in demand for security authentication, light fidelity, optical storage, laser communication, display technologies, and optical information processing.^[^ ^19^ ^]^ Combined with the picturesque characteristic of organic semiconductor molecules, including tailormade molecular structure, the low cost large‐area fabrication, strong light–matter interactions, and compatibility with plastic substrates,^[^ ^20^ ^]^ the organic NIR emitters have drawn considerable research attention in both fundamental importance and practical implication. As shown in Figure 1b, there are much different excited states with a distinct absorption process, resulting in a complex excited process for organic small molecules. In addition, the energy gap law generates an intrinsic fast nonradiative decay, leading to a main challenge for the high PL quantum efficiency of organic small molecules with a narrow bandgap. Up to date, the rational molecular design and synthesis of the HLCT compounds, TADF materials, CT cocrystals, neutral π radicals, and ESIPT‐active materials with attractive NIR emission have undergone great progresses, as given in Figure 1c–e.

### 2.1 Hybridized Local and Charge Transfer (HLCT) Compounds

**Figure 2:**
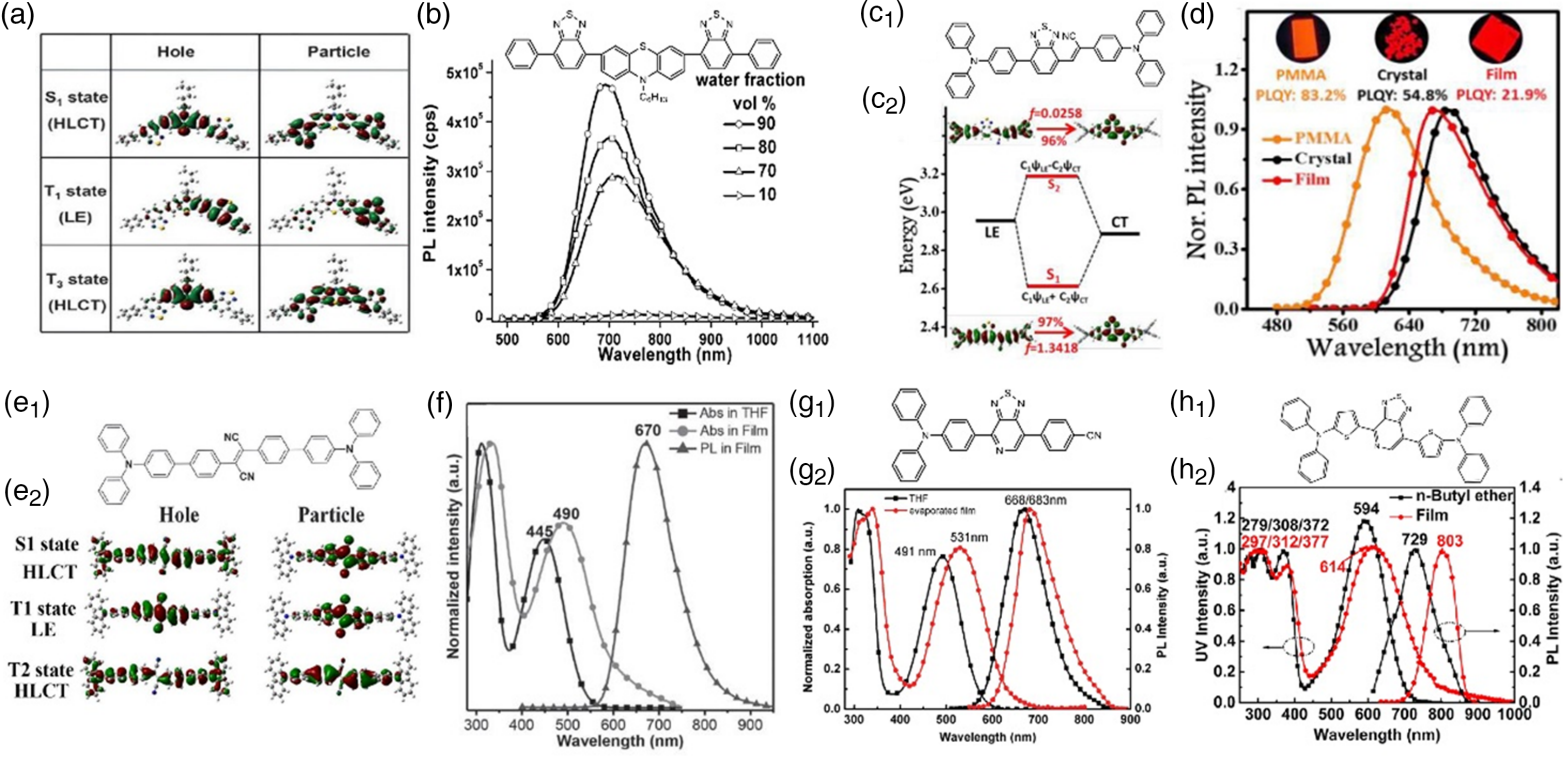
a) Molecular structure of TZ–BZP, and its natural transition orbitals for *S*_1_, *T*_1_, and *T*_3_. b) PL spectra of PTZBZP (10 mm) in THF/water mixtures with different water fractions. Reproduced with permission.^[^ ^24^ ^]^ Copyright 2014, Wiley‐VCH. c_1_) Molecular structure of BTA–TPA. c_2_) The NTOs from *S*_0_ to *S*_1_ and *S*_2_, where *f* represents the oscillator strength. d) The steady‐state PL spectra of BTA–TPA. Reproduced with permission^[^ ^25^ ^]^ Copyright 2020, Royal Society of Chemistry. e_1_) Molecular structure of TPATCN. e_2_) The nature transition orbitals for *S*_1_, *T*_1_, and *T*_2_. f) The UV–vis spectra in diluted THF solution (concentration: 1 × 10^−5^ mol L^−1^) and the UV–vis and PL spectra in vacuum‐evaporated film (excitation wavelength (*λ*_ex_): 490 nm). Reproduced with permission.^[^ ^26^ ^]^ Copyright 2015, Wiley‐VCH. g_1_) Molecular structure of *p*‐TPA‐PT‐CN. g_2_) The UV–vis spectra and PL spectra of *p*‐TPA‐PT‐CN in THF and evaporated film. Reproduced with permission.^[^ ^27^ ^]^ Copyright 2017, Royal Society of Chemistry. h_1_) Molecular structure of DTPS–PT. h_2_) The UV−vis spectra and PL spectra of DTPS–PT in *n*‐butyl ether solution and evaporated film. Reproduced with permission.^[^ ^28^ ^]^ Copyright 2019, American Chemical Society.

The polar D–A π‐conjugated configuration with a powerful intramolecular CT interaction demonstrates a large dipole for the narrow bandgap, which has been verified as a serviceably adopted design strategy for the NIR‐emissive molecules.^[^ ^21^ ^]^ However, the forbidden electronic transitions of the CT state will terribly quench fluorescence feature.^[^ ^22^ ^]^ It is well known that the LE state generally gives rise to a large orbital overlap, which is favorable for high PLQYs.^[^ ^23^ ^]^ The HLCT excited state combined with LE and CT states could significantly lessen the energy gap, benefiting a high PLQY, which is a promising building block for highly efficient NIR emission. Accordingly, Ma and co‐workers applied the phenothiazine electron donor and benzothiadiazole electron acceptor to design and synthesize a butterfly‐shaped D–A‐type fluorophore of PTZ–BZP with a typical HLCT‐excited state.^[^ ^24^ ^]^ From the S_1_ state of PTZ–BZP (**Figure** **2**a), an excellent balance between spatial separation and orbital overlap was observed in the hole and particle NTOs. Meanwhile, the well‐separated orbitals resulted in a remarkable CT feature with a big dipole moment. In addition, the LE character originating from certain orbital overlaps benefits the high radiative transition rate. The coexistence of LE and CT components clarified the formation of the HLCT state, leading into strong NIR fluorescence with a high PLQY of 16% of PTZ–BZP (Figure 2b). It is well known that the discrete dimeric packing is favorable for the lengthened PL wavelength. The HLCT emitter of benzothiadiazole‐triphenylamine (BTA‐TPA) was rationally designed and prepared, which could display discrete dimeric packing. Figure 2c displays an outstanding balance between the orbital overlap and the spatial separation in the particle and hole NTOs.^[^ ^25^ ^]^ The distinguishable separated orbitals and sufficient orbital overlap were respectively attributed to the CT feature and LE characteristic, suggesting the achievement of the HLCT state. Due to the discrete dimeric packing, BTA‐TPA crystal achieved NIR fluorescence peak at 690 nm with a high PLQY of 54.8%, while the BTA–TPA film has deep‐red emission with a high PLQY of 21.9% (Figure 2d).

Furthermore, 2,3‐bis(4′‐(diphenylamino)‐[1,1′‐biphenyl]‐4‐yl) fumaronitrile (TPATCN) with the D–A–D‐type molecular structure was rationally designed and prepared, which holds a relatively a high orbital overlap of the LE state and a big dipole moment of the CT state, suggesting the HLCT state (Figure 2e).^[^ ^26^ ^]^ As shown in Figure 2f, the absorption maximum at 445 nm is originated from a low‐energy intramolecular CT transition, as well as the redshift emission of TPATCN solution with the increasing solvent polarity evidenced the CT character of the excited state. Typically, the TPATCN showed deep‐red emission at 670 nm in the film state and NIR emission at 785 nm in THF solution (Figure 2f). The acceptor of pyridal[2,1,3]thiadiazole (PT) with pyridine heterocycle and the donors of cyanosubstituent (CN) and triphenylamine (TPA) were used to design and synthesize the high‐efficiency NIR emitter of *p*‐TPA‐PT‐CN with the HLCT state (Figure 2g).^[^ ^27^ ^]^ The introduced pyridine nitrogen significantly reduced the steric hindrance effect between PT heterocycle and the adjacent TPA donor segment, increasing the planarity and intensity mixing of HLCT state. As shown in Figure 2g, the long‐wavelength absorption peaks at 491 and 531 nm were indexed according to the intramolecular CT transition of *p*‐TPA‐PT‐CN, resulting in NIR emission with PL peak at 685 nm. Because of higher planarity of the molecular structure, 5,5′‐([1,2,5]thiadiazolo[3,4‐c]pyridine‐4,7‐diyl)bis(N,N‐diphenylthiophen‐2‐amine) (DTPS‐PT) demonstrated enhanced piconjugation and hybridization between the CT state and LE states.^[^ ^28^ ^]^ Thus, DTPS‐PT showed NIR emission of around 803 nm with a high PLQY of 79% in tetrachloromethane (Figure 2h).

### 2.2 TADF Materials

**Figure 3:**
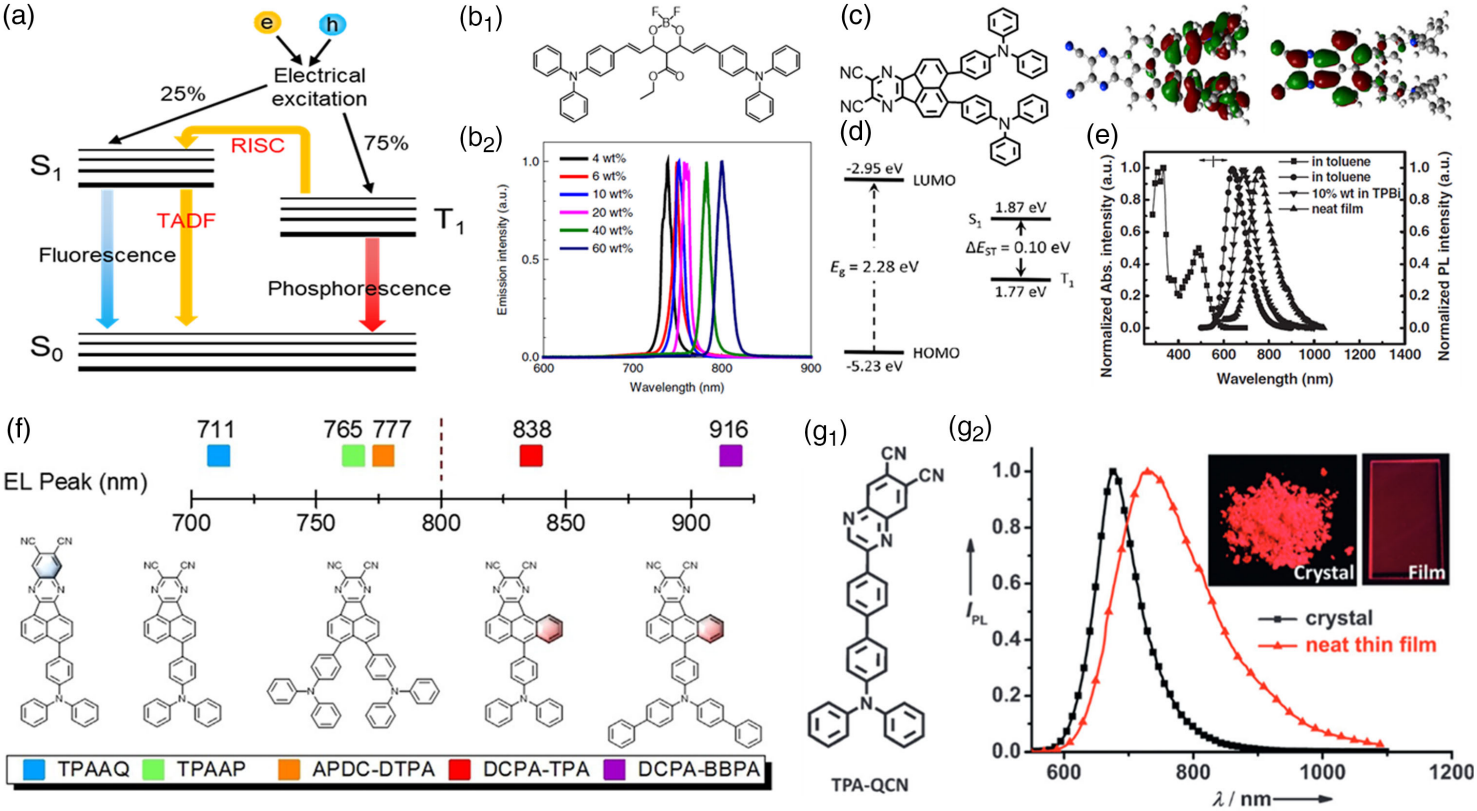
a) Energy diagram of a conventional organic molecule. Reproduced with permission^[^ ^29^ ^]^ Copyright 2012, Springer Nature. b_1_) The molecular structure of a boron difluoride curcuminoid complex. b_2_) The corresponding PL spectra of CBP blend films doped with various doping concentrations. Reproduced with permission^[59b]^ Copyright 2019, American Chemical Society. c) The molecular structure, HOMO, and LUMO distributions of APDC–DTPA. d) The calculated HOMO, LUMO, singlet (*S*_1_), and triplet (*T*_1_) energy levels of APDC–DTPA. e) Absorption and PL spectra of APDC–DTPA. Reproduced with permission.^[^ ^36^ ^]^ Copyright 2017, Wiley‐VCH. f) Molecular structures and emission peaks of TPAAQ, TPAAP, APDC–DTPA, DCPA–TPA, and DCPA–BBPA. Reproduced with permission.^[^ ^39^ ^]^ Copyright 2020, Wiley‐VCH. g_1_) Molecular structure of TPA–QCN. g_2_) The corresponding PL spectra of TPA–QCN. Inset: Images of crystal and neat thin film under UV irradiation (*λ*_ex_ = 365 nm). Reproduced with permission.^[^ ^41^ ^]^ Copyright 2017, Wiley‐VCH.

Generally, there are 25% *S*_1_ excitons and 75% *T*_1_ excitons produced in the organic‐emissive semiconductors, as shown in **Figure** **3**a.^[^ ^29^ ^]^ The conventional fluorescence materials could just utilize 25% *S*_1_ excitons for emission, which promotes sciencers to increase the luminous efficiency to meet the satisfyingly commercial parameters via utilizing nonradiative triplet excitons. Moreover, combining with heavy metals (such as Os, Ir, Pt, etc.), the formed organic metal complex could enhance the spin–orbit coupling for effectively utilizing *T*_1_ excitons via a phosphorescence radiative transition.[^2^, ^8^, ^30^] In contrast, organic TADF emitters could utilize the triplet excitons for high photoluminescence efficiency via an effective RISC process from the nonradiative *T*_1_ state to a radiative *S*_1_ state at room temperature.^[^ ^12^, ^31^ ^]^ The small energy gap (Δ*E*_ST_) between the *T*_1_ and *S*_1_ states is of ≤100 meV and is necessary for the RISC process.^[^ ^32^ ^]^ The intramolecular CT interaction within systems containing spatially separated donor and acceptor moieties is favorable for the RISC process, leading to enhanced photoluminescence efficiency. It means that most TADF emitters show great advantages in high‐efficiency emission. Adachi and co‐workers demonstrated a TADF emitter of a boron difluoride curcuminoid derivative with a D–A–D structure, which contained an acetylacetonate boron difluoride acceptor unit and two triphenylamine donor groups (Figure 3b).^[^ ^33^ ^]^ Furthermore, this TADF emitter exhibits NIR emission with the maximum emission wavelength from 700 to 780 nm at room temperature.

The electronic donor and accepter linked by the phenylene linker (Ph) were widely employed to modify the intermolecular CT interaction, resulting in a larger dipole moment for the redshifted emission compared with simple D–A structure.^[^ ^13^, ^34^ ^]^ In addition, the Ph group plays a helpful effect for the frontier molecular orbitals with a rational overlap, providing faster radiative rate and lower Δ*E*_ST_, resulting in an intense NIR emission.^[^ ^35^ ^]^ Based on this consideration, our group utilized acenaphtho[1,2‐b] pyrazine‐8,9‐dicarbonitrile (APDC) with stronger electron‐withdrawing ability as the acceptor to couple with two diphenylamine (DPA) donor units, forming NIR emitters of APDC–DTPA with a wedge‐shaped D–π–A–π–D structure (Figure 3c).^[^ ^36^ ^]^ The obvious overlap between the HOMO and the LUMO of APDC–DTPA supplies a small Δ*E*_ST_ of 0.14 eV (calculated 0.10 eV), which facilitates the RISC for strong emission. Due to the narrow bandgap of 2.28 eV, the APDC–DTPA displayed NIR fluorescence with an emission PL peak of 756 nm (Figure 3e). It is illuminated that the strong CT state could decrease the Δ*E*_ST_ and stabilize the excited states, realizing the highly efficient emission in NIR region for TADF materials.^[^ ^37^ ^]^ Furthermore, triphenylamine (TAP) and 8‐(4‐(di([1,1′‐biphenyl]‐4‐yl)amino)phenyl) (BBPA) were also introduced into APDC, in which the intramolecular CT interaction and the dipole moment of DCPA–BBPA are stronger than those of DCPA–TPA. Thus, DCPA–BBPA exhibits impressively further redshifted NIR emission with an electroluminescence (EL) peak at 916 nm, in contrast with EL peak at 838 nm of DCPA‐TPA. It indicates that the molecular structure plays a crucial influence for the optoelectronic properties of the organic semiconductors. Qiao and co‐workers also employed the strong intermolecular CT characteristics to design and prepare the highly efficient TADF materials with NIR emission.^[^ ^38^ ^]^ As described in Figure 3f, TPAAQ and TPAAP were prepared based on the strong electron‐donating donor of triphenylamine (TPA) combined with the rigid and powerful electron‐drawing acceptors of acenaphtho[1,2‐b]quinoxaline‐8,9‐dicarbonitrile (AQ) and acenaphtho[1,2‐b]pyrazine‐8,9‐dicarbonitrile (AP), respectively.^[^ ^38^ ^]^ Furthermore, TPAAQ and TPAAP exhibited the NIR emission with the PL peaks at 711 and 765 nm. Meanwhile, the NIR‐emissive DCPA–BBPA as well as DCPA–TPA were also prepared via functionalizing anthryleno[1,2‐b]pyrazine‐2,3‐dicarbonitrile (DCPA) acceptor with aromatic amine donors.^[^ ^39^ ^]^ The intensity intramolecular CT interaction between the amine donor and the DCPA acceptor facilitates the formation of large dipole moments, realizing the outstanding NIR fluorescence with emission PL peaks of 838 and 916 nm for DCPA–TPA and DCPA–BBPA.

Furthermore, the fine molecular design and supramolecular structure modulation of the TADF emitter provide an alternative approach for the tunable emission color.^[^ ^40^ ^]^ The electron acceptor quinoxaline‐6,7‐dicarbonitrile (QCN) and electron donor triphenylamine (TPA) were used to obtain the TADF emitter of TPA–QCN with a D–π–A‐conjugated framework.^[^ ^41^ ^]^ The relatively steric effect of TPA plays an important role in reduced dipole···dipole interaction, showing enhanced emission. Due to the crooked π‐conjugation geometry and the intermolecular CT interaction, TPA–QCN molecules aggregate into the edge‐to‐edge stacking mode instead of face to face or π···π stacking, resulting in redshift emission and considerably weaker emission quenching. Therefore, the TPA–QCN single crystal displays strong deep‐red luminescence with a PLQY of 38% and an emission PL peak at 677 nm (Figure 3g). In contrast, TPA–QCN molecules in the film phase adopt more dense packing form relative to the crystal phase, which displays shorter intermolecular contacts and results in a larger redshift of the emission maximum. Therefore, the TPA–QCN neat thin films exhibit NIR emission with a PL peak at 733 nm and a PLQY of 21%. For substantial quinoidal character and greater electron delocalization, benzo(1,2‐c:4, 5‐c′)bis((1,2,5)thiadiazole) (BBTD) was chosen as the electron acceptor to form the TADF‐type emitting materials of 4,8‐Bis[4‐(*N,N*‐diphenylamino) phenyl]‐BBTD, 4,8‐Bis[4‐(*N*‐phenyl‐*N*‐(4‐methylphenyl)amino)phenyl]‐BBTD and 4,8‐Bis[4‐(*N*‐phenyl‐*N*‐(1‐naphthyl)amino)phenyl]‐BBTD and 4,8‐Bis[5‐(*N,N*‐diphenylamino)‐2‐thiophene]‐BBTD. Due to the nonplanar, bulky structure of the donor moiety and the restricted rotational motions of the twisted triarylamine moiety in the solid state, the nonradiative process in these TADF‐type emitting materials was suppressed, leading to intense PL above 1000 nm.^[^ ^42^ ^]^ The J‐aggregates with strong intermolecular CT interaction were used to design and prepare high‐efficiency NIR TADF of (3‐(4‐(diphenylamino)phenyl)acenaphtho[1,2‐b]pyrazino[2,3‐e]pyrazine‐9,10‐dicarbonitrile) (TPAAZ) with the PL peak beyond 1000 nm.^[^ ^43^ ^]^ Due to the formation of the intermolecular CT aggregates, the TPAAZ neat films achieved NIR‐II region emission with the PL peak at 1009 nm.

### 2.3 Organic CT Cocrystals

The organic electronic donor and acceptor molecules assemble into highly ordered superstructures via the intermolecular CT interactions, forming organic CT cocrystals.^[^ ^44^ ^]^ Due to the novel CT interactions and the molecular packing modes, the organic CT cocrystals not only hold the chemicophysics properties of the individual components, but also generate the unusual optoelectronic properties, which are absent in their constitute components.^[^ ^45^ ^]^ Therefore, great efforts have been recently devoted to investigate the organic CT cocrystals toward the desired optoelectronic functions, including ferroelectricity,^[^ ^46^ ^]^ optical waveguide,^[^ ^40^ ^]^ room‐temperature phosphorescence,^[^ ^47^ ^]^ photothermal conversion,^[^ ^48^ ^]^ and ambipolar charge transport.^[^ ^49^ ^]^ Notably, the CT interaction between the electron donor and acceptor plays a significant role for π‐electron delocalization, which contributes to band engineering for orbital hybridization. Furthermore, the donor HOMO and acceptor LUMO have a larger contribution to the HOMO and LUMO of the organic CT cocrystals. It is suggested that the organic CT cocrystal demonstrates a new energy structure with a low energy gap for the distinct redshift in absorption or emission.^[^ ^50^ ^]^ However, the excitons with the low bandgap of CT complex easily return to the ground state through a competitive nonradiative decay channel,^[2b]^ leading to a significant challenge in the successful exploration of new NIR emitters.

**Figure 4:**
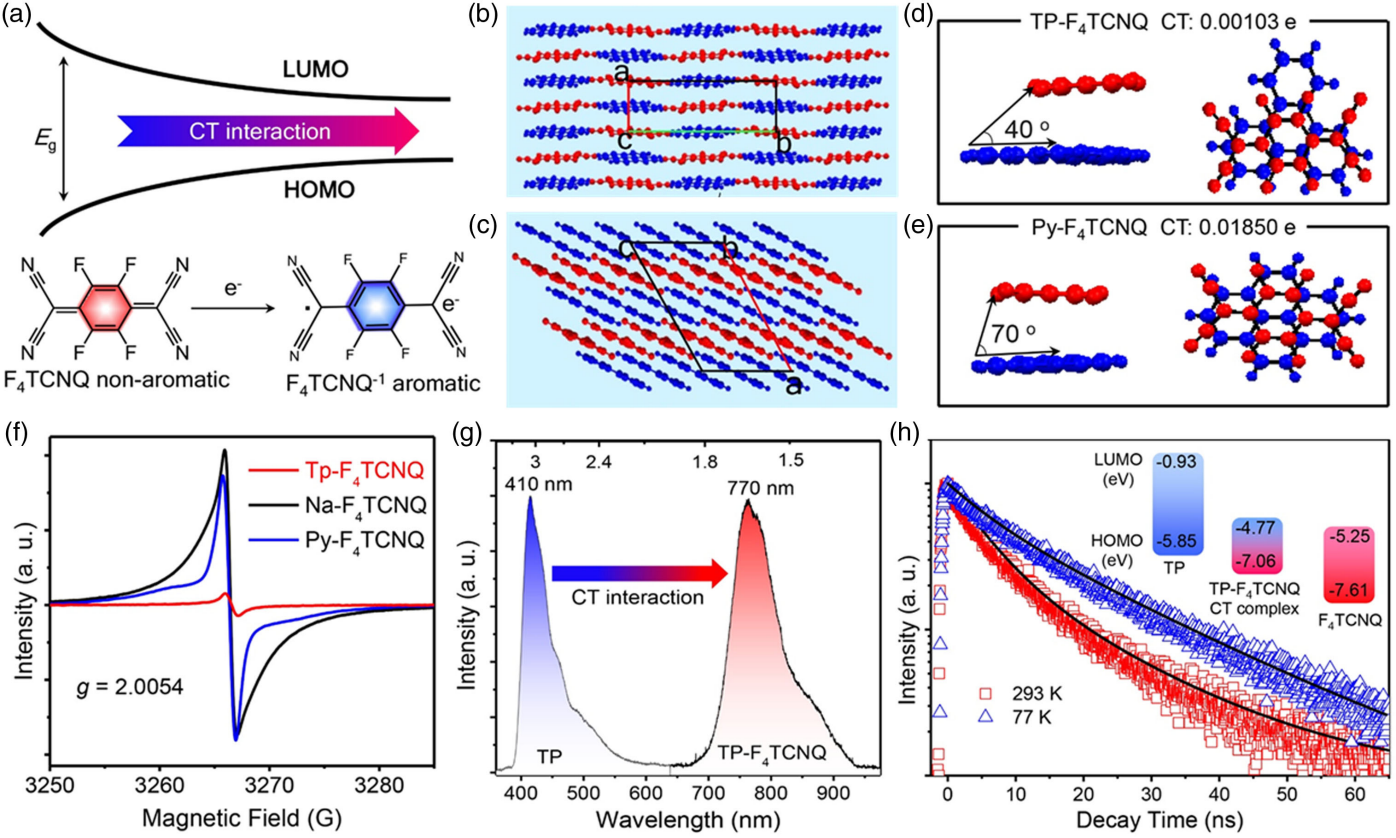
a) Schematic diagrams of energy evolution of the CT cocrystal and the TCNQ reduction yields a stable aromatic radical anion. The molecular packing mode of b) Py–F_4_TCNQ and c) TP–F_4_TCNQ cocrystals. The counter pitch angles and the CT amount of d) Py–F_4_TCNQ and e) TP–F_4_TCNQ cocrystals. f) ESR spectra of CT cocrystals. g) PL spectra of TP and TP–F4TCNQ. h) The typical PL decay profile at different temperatures. Inset: The calculated energy diagrams. Reproduced with permission.^[^ ^52^ ^]^ Copyright 2022, Wiley‐VCH.

Anisotropic molecular packing mode could directly decide the optoelectronic features of organic CT complex.^[^ ^51^ ^]^ Drawing inspiration from this point, our group first applied the rational modulation of the molecular packing modes to tailor the photophysical properties of organic CT cocrystals, realizing the desired NIR emitters.^[^ ^52^ ^]^ As given in **Figure** **4**a, the CT interaction promotes the convention of F_4_TCNQ molecular structure from the neutral quinonoid form state to aromatic form state after accepting an electron, generating an electronic coupling and a narrow energy gap for longer wavelength of absorption or emission. In addition, strong CT interaction is desired for increased radical concentration, resulting in fluorescence quenching.^[^ ^53^ ^]^ Pyrene (Py) and triphenylene (TP) were employed as electronic donors to design and prepare the CT cocrystals after combining with electronic acceptor of F_4_TCNQ via the CT interaction. The Py–F_4_TCNQ cocrystal displays a mixed packing mode with strong π–π overlapping (Figure 4b), while TP and F_4_TCNQ molecules display a suitable slippage compared with typical face‐to‐face packing, forming a segregated stacking mode for TP–F_4_TCNQ cocrystals (Figure 4c).^[^ ^40^, ^54^ ^]^ As given in Figure 4d,e, the counterpitch angles between donor and acceptor for Py–F_4_TCNQ and TP–F_4_TCNQ cocrystals are 70° and 40°, respectively, which are conducive to the different molecular packing modes. Furthermore, the low counterpitch angle of TP–F_4_TCNQ cocrystal results in a calculated CT degree of 0.00103e, which is lower than that of 0.01850e for Py–F_4_TCNQ cocrystal. It is indicated that the CT interaction between Py and F_4_TCNQ molecules is stronger than that between TP and F_4_TCNQ molecules. It was also confirmed by the higher electron spin resonance (ESR) intensity of Py–F_4_TCNQ cocrystal (Figure 4f) than that of TP–F_4_TCNQ cocrystal. The small counter pitch angle (40°) between F_4_TCNQ and TP molecules and the low CT degree (0.00103e) are conducive to changing the forbiddance of CT‐state electron transitions, realizing effective emission in NIR region. Except for the main nonradiative process of VR, there are appreciable quantity excitons at CT_1_ state that return to the ground CT_0_ state. Thus, TP–F_4_TCNQ cocrystals displayed the NIR emission beyond 760 nm and a PLQY of 5.4%, as illustrated in Figure 4g. The single exponential of the corresponding PL lifetime indicates the NIR emission of TP–F_4_TCNQ cocrystals coming from the single‐exited CT state, rather than the existence of the RISC for TADF (Figure 4h).

### 2.4 Neutral Π‐Radicals

**Figure 5:**
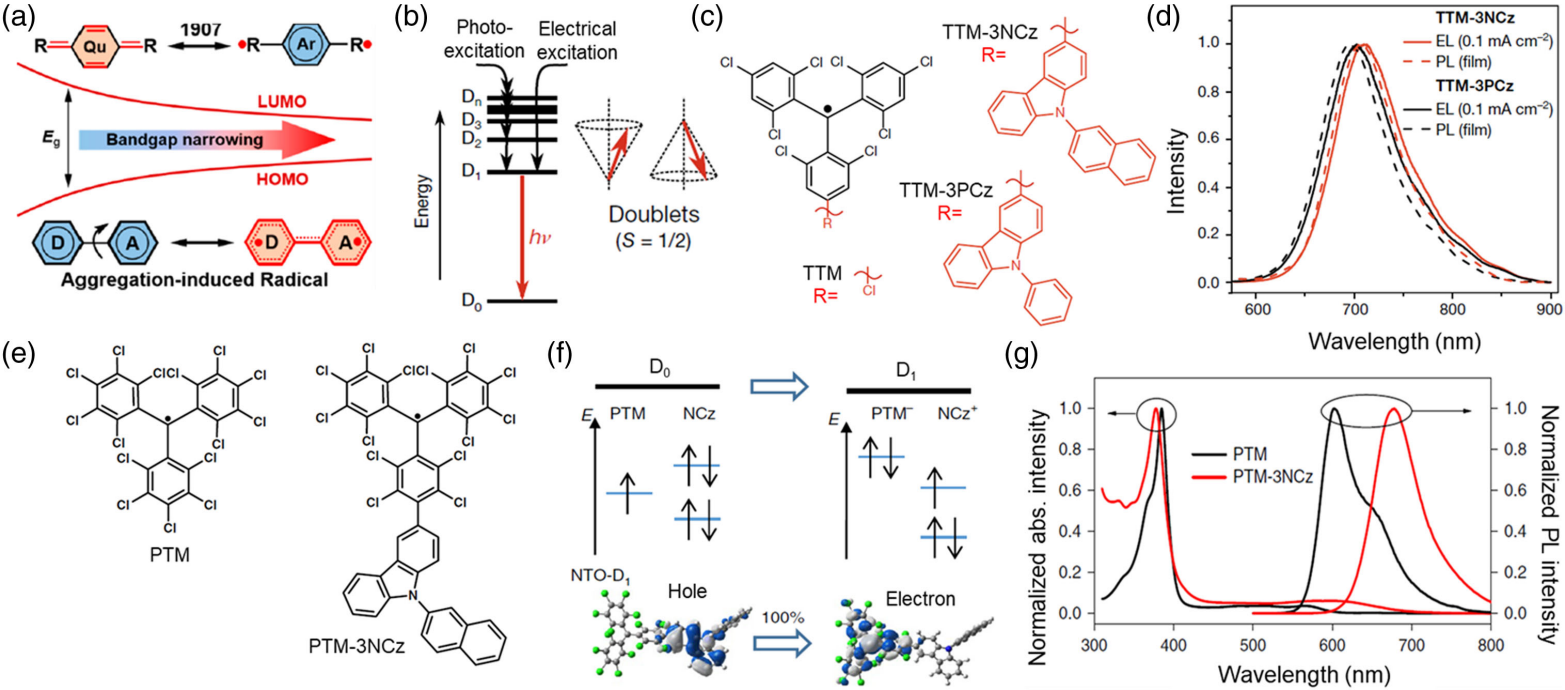
a) Narrow bandgap of organic semiconductors with open‐shell singlet states. Reproduced with permission.^[56b]^ Copyright 2021, American Chemical Society. b) Doublet emission following photo‐ and electrical excitation. The illustration on the right indicates the electron spin vector representation for doublets. c) Molecular structures of TTM, TTM–3NCz, and TTM–3PCz. d) PL (dotted lines) spectrum for TTM–3NCz (red) and TTM–3PCz (black). Reproduced with permission.^[^ ^57^ ^]^ Copyright 2018, Springer Nature. e) Molecular structures of PTM and PTM–3NCz. f) Schematics of the electronic transition process between the ground state (*D*_0_) and the first excited state (*D*_1_) in PTM–3NCz. The two colors used for the electron wavefunctions distinguish the wavefunction phases; the white, grey, blue, and green spheres in the chemical structures represent H, C, N, and Cl atoms, respectively. g) Absorption and PL spectra of PTM and PTM–3NCz radicals in cyclohexane. Reproduced with permission.^[^ ^58^ ^]^ Copyright 2019, Springer Nature.

The organic radical molecules with unique electronic structures demonstrate novel luminescence properties, which are conducive to their potential lighting applications.^[^ ^55^ ^]^ As shown in **Figure** **5**a, after accepting electrons by the organic semiconductors, the corresponding neutral quinonoid form state will change into the aromatic form state, increasing the active radicals and the unpaired electrons for a narrow bandgap for redshift absorption or emission.^[^ ^56^ ^]^ Furthermore, the CT process of the organic semiconductors with the D–A structure could also generate the active radicals and unpaired electrons with a narrow bandgap. It indicates that the organic radical molecules have the great potential application in NIR emission. Generally, there is a singly occupied molecular orbital (SOMO) with an overall spin‐1/2 doublet in the ground state for the organic radical emitters (Figure 5b), which is favorable for emission via avoiding the forbidden state of the triplet state.^[^ ^57^ ^]^ For the doublet‐ground‐state (D_0_) molecules with D–A structure, the photoexcitation will generate doublet excited states (D_*n*_), leading to the spin‐allowed emission of fluorescence originating from the lowest‐lying D_1_.^[^ ^57^ ^]^ It is well known that tris(2,4,6‐trichlorophenyl)methyl (TTM) is a typical organic radical molecule (Figure 5c). To obtain a narrow bandgap for NIR emission, 3‐substituted‐9‐phenyl‐9 H‐carbazole (3PCz) and 3‐substituted‐9‐(naphthalen‐2‐yl)‐9 H‐carbazole(3NCz) were used as the electronic donors for the construction of the D–A structure via incorporating with the electronic acceptor TTM radical groups, forming TTM‐3PCz and TTM‐3NCz emitters. Their first excited states demonstrate a strong CT character, leading to large spatial separation with a small overlap between TTM‐centered SOMO and 3NCz/3PCz‐centred HOMO. Therefore, both TTM‐3PCz and TTM‐3NCz display NIR emission with PL peak at 710 nm (Figure 5d), which is derived from the same electronic transition (D_1_ → D_0_). To enhance the PLQY and emission stability, Li and co‐workers designed a donor–acceptor‐neutral radical (Figure 5e) that depended on an electron‐poor perchlorotriphenylmethyl (PTM) radical moiety incorporated with an electron‐rich group of 9‐(naphthalene‐2‐yl)‐9H‐carbazole (NCz).^[^ ^58^ ^]^ The PTM radical segment has a regional π–π* excitation, as well as PTM‐3NCz, which displays a representative CT excitation, as verified by the theoretical natural transition orbitals (Figure 5f). The natural radicals of PTM and PTM‐3NCz display an maximum absorption peak at ≈380 nm in the absorption spectra (Figure 5g). Importantly, an additional peak at 607 nm is also found in PTM‐3NCz, corresponding to the CT excitation. Moreover, the PTM‐3NCz radical demonstrated a significant redshift in contrast to that of PTM, leading to desired NIR emission at 680 nm (Figure 5g).

### 2.5 Excited‐State Intramolecular Proton Transfer (ESIPT)‐Active Materials

**Figure 6:**
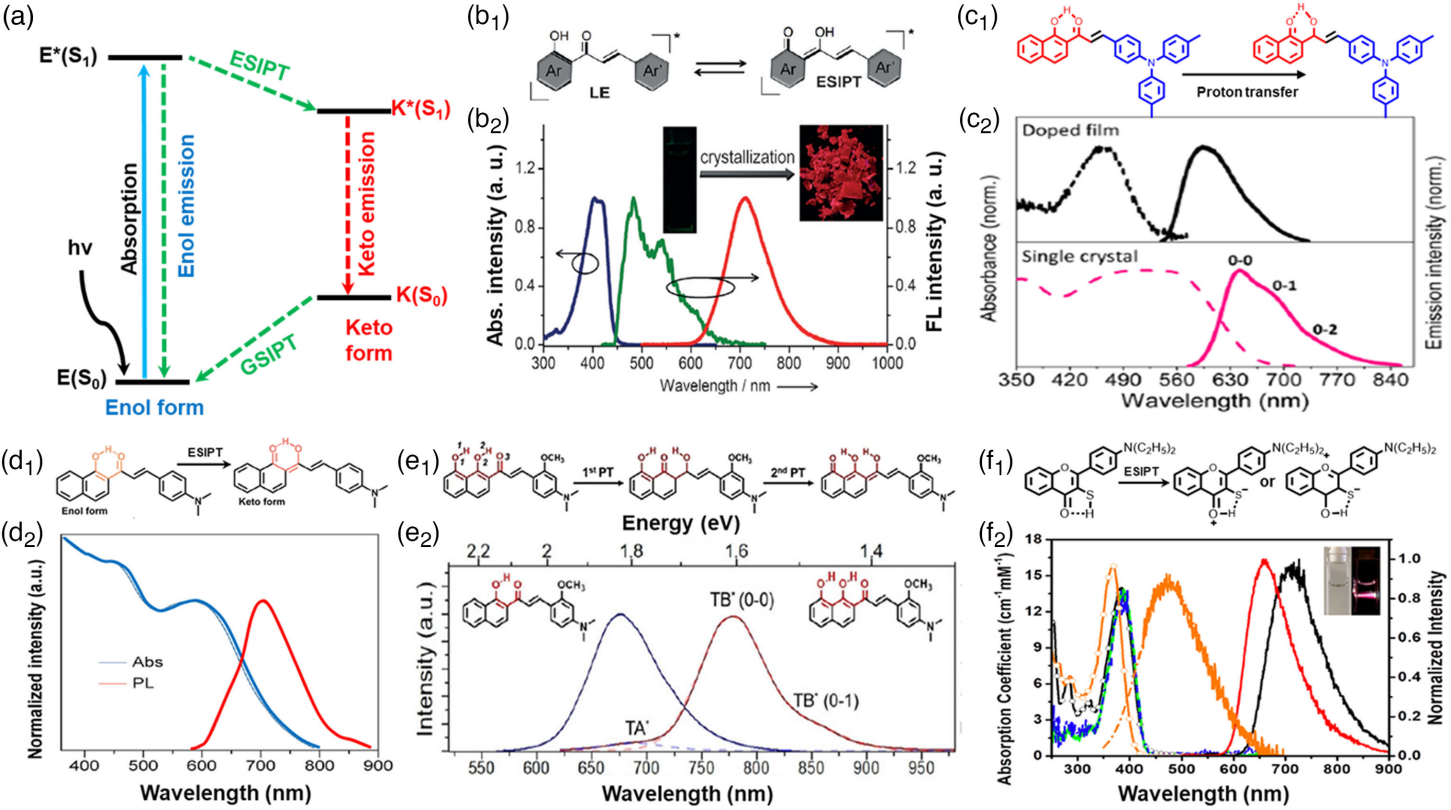
a) Four‐level ESIPT process. Reproduced with permission.^[^ ^60^ ^]^ Copyright 2016, Royal Society of Chemistry. b_1_) The proposed ESIPT of HDMAC. b_2_) The absorption and emission spectra in *n*‐hexane and crystal of HDMAC. Reproduced with permission.^[^ ^62^ ^]^ Copyright 2015, Wiley‐VCH. c_1_) The proposed ESIPT of DPHP from the enol form to the keto form. c_2_) The absorption (dotted line) and emission (solid line) spectra of DPHP‐doped film (black color) and single crystal (red color). Reproduced with permission.^[^ ^63^ ^]^ Copyright 2015, American Chemical Society. d_1_) Schematic of ESIPT process. d_2_) The diffuse reflectance absorption (blue line) and photoluminescence spectra (red line) of the DMHC single crystal. Reproduced with permission.^[^ ^66^ ^]^ Copyright 2020, Elsevier. e_1_) Diagram of the cascaded proton transfer of the DDMP system. e_2_) The PL spectra of the DMHP single‐crystal nanowires (blue line) and the DDMP single‐crystal nanowires (red line). Reproduced with permission.^[^ ^9^ ^]^ Copyright 2021, Wiley‐VCH. f_1_) The proposed ESIPT of 3NTF, forming a zwitterionic‐type tautomer with two possible canonical structures. f_2_) The absorption (circle and line) and emission (solid line) spectra of 3NTF (black) and 3MeNTF (orange) in cyclohexane at 298 K. Reproduced with permission.^[^ ^69^ ^]^ Copyright 2021, American Chemical Society.

The photochemical process of ESIPT is the proton transfer from sulfhydryl or hydroxyl groups to carbonyl group induced by intramolecular hydrogen bonding under excitation, producing a new molecular configuration of a tautomer species (keto form).^[^ ^59^ ^]^ The organic molecules with the ESIPT property exhibit four electronic levels from normal to tautomer forms with excitation, which could supply a desired multilevel energy system (**Figure** **6**a).^[^ ^60^ ^]^ Notably, the keto form displays a narrow bandgap for the longer‐wavelength emission without a distinct reabsorption compared with the enol form.^[^ ^61^ ^]^ It is suggested that the ESIPT process is useful for the redshift emission, especially NIR emission. Professor Wang and co‐workers synthesized 3‐[4‐(dimethylamino)phenyl]‐1‐(2‐hy‐droxyphenyl)prop‐2‐en‐1‐on (HDMAC) based on 2′‐hydroxychalcone with the ESIPT property.^[^ ^62^ ^]^ As shown in Figure 6b, the HDMAC single crystal displays NIR emission with an emission maximum of over 710 nm and a high PLQY of 32%. Due to the ESIPT process, the HDMAC single crystal also displays a large Stokes shift. Furthermore, triphenylamine with a propeller shape‐like molecular geometry was introduced into 2′‐hydroxychalcone, forming (*E*)‐3‐(4‐(di‐ptolylamino)phenyl)‐1‐(1‐hydroxynaphthalen‐2‐yl)prop‐2‐en‐1‐one (DPHP).^[^ ^63^ ^]^ Under excitation, the intramolecular hydrogen bonding induces the proton transfers from hydroxyl group to carbonyl group for a typical ESIPT process (Figure 6c). Furthermore, the DPHP‐doped film exhibits absorption maximum at 461 nm and a PL maximum at 596 nm. In contrast, the DPHP single crystal demonstrates a strong NIR emission with a broad PL spectrum in NIR region from 643 to 756 nm, respectively, which is attributed to the strong π–π interaction^[^ ^64^ ^]^ and the aggregation‐induced emission.^[^ ^65^ ^]^ Due to the restriction of intramolecular rotations, PLQYs of the DPHP doped film and DPHP single crystal are ≈10.3% and 15.2%, which are larger than that of the solution (≈0.23%).

It is well known that the extension of the conjugation length and the D–A molecule skeleton is beneficial for the lower energy gap, leading to redshift emission.^[^ ^18^ ^]^ Given these considerations, an another ESIPT molecule of (*E*)‐3‐(4‐(dimethylamino)phenyl)‐1‐(1‐hydroxynaphthalen‐2‐yl)prop‐2‐en‐1‐one (DMHC) was rationally designed and successfully synthesized.^[^ ^66^ ^]^ The absorption spectrum of DMHC solution (cyclohexane) exhibits the peak at 450 nm, as verified in Figure 6d. In addition two peaks at 530 and 610 nm in the corresponding PL spectrum respectively contribute to the enol form and the keto form. Meanwhile, the absorption and PL‐centered peaks of the DMHC single crystal are 550 and 700 nm, which are due to the ESIPT process. Similarly, the ESIP molecules show NIR emission, such as 4‐dimethylamino‐2′‐hydroxychalcone with deep‐red and NIR emission at 600–750 nm,^[^ ^67^ ^]^ (*E*)‐3‐(4‐(dimethylamino)‐2‐methoxyphenyl)‐1‐(1‐hydroxynaphthalen‐2‐yl)prop‐2‐en‐1‐one (DMHP) with NIR emission at 720 nm,^[^ ^68^ ^]^ 2‐(4‐(diethylamino)phenyl)‐3‐mercapto‐4H‐chromen‐4‐one with the NIR emission at 710 nm in cyclohexane.^[^ ^69^ ^]^ The cascaded occurrence of the ESIPT process is regarded as a promising approach to obtain a more narrow bandgap for NIR emission. Inspired by this molecular design, our group further finely prepared the cascaded ESIPT‐active molecule of (*E*)‐1‐(1,8‐dihydroxynaphthalen‐2‐yl)‐3‐(4‐(dimethylamino) −2‐methoxyphenyl)prop‐2‐en‐1‐one (DDMP).^[9a]^ Under excitation, the DDMP exhibits two adjacent intramolecular hydrogen bonds, leading to the cascaded occurrence of ESIPT process, as verified in Figure 6e. Compared with the deep‐red emission (670 nm) of the DMHP nanowire with the simple ESIPT process, the DDMP nanowires display an NIR emission (PL peak:778 nm) with a PLQY of 1.5%, which is ascribed to the cascaded occurrence of ESIPT process. In additional, the proton transfers based on the intramolecular hydrogen bonding between sulfhydryl groups and carbonyl are also favorable for the ESIPT process based on 2‐(4‐(diethylamino)phenyl)‐3‐mercapto‐4H‐chromen‐4‐one (3NTF).^[^ ^69^ ^]^ Upon photoexcitation, 3NTF exhibits charming NIR emission with a maximized PL peak at 710 nm and an anomalously large Stokes shift of 12 230 cm^−1^ in cyclohexane (Figure 6f).

## 3 Conclusion and Outlook

Immense efforts have been devoted to developing novel organic semiconductors with desired NIR emission via the rational molecular design, which is attributed to their unique advantages in contrast to their visible emissive counterparts. The organic NIR emitters have been proved to be promising candidates for optoelectronic and imaging applications in chemistry‐materials, physics, and biology, such as communication, night vision, and biomedical sensors. Such comprehensive and fundamental applications encourage the researchers to develop a design strategy and synthesis technology toward organic emitters with an increased PLQY or a larger emissive peak in the NIR region.^[^ ^70^ ^]^ Nevertheless, NIR emitting emitters usually suffer the aggregation‐caused quenching (ACQ) effect originating from a rigid plane conjugation or a strong donor/acceptor (D/A) configuration of NIR molecules,^[^ ^71^ ^]^ as well as the dramatically decreased luminescence efficiency as the PL wavelength enhances following an energy gap law.^[9a]^

It is well known that the conventional fluorescent chromophores can just utilize singlet excitons for NIR emission, leading to low emission efficiency and faring from the practical satisfaction for commercialization application. Neutral π‐radicals, TADF, and HLCT materials show great advantages in utilizing the remaining excitons in triplet states for increasing the emission efficiency via adjusting the molecular structure conversion between the quinoid and aromatic form or tuning the CT interactions. Furthermore, these organic NIR materials were successfully applied in organic light‐emitting diodes (OLEDs) with high performance. For example, OLEDs based on a radical emitter of TTM–PPTA show NIR emission with a maximal external quantum efficiency of 5.3%.^[^ ^58^ ^]^ The ESIPT‐active NIR materials draw great attention in supplying narrowing of the bandgap for NIR emission with large Stokes shifts, especially supplying the intrinsic four‐level system for achieving the organic NIR laser with low threshold in the solid state.^[^ ^72^ ^]^ The cocrystal engineering was regarded as a vital functional material design strategy, which supplies great opportunities for precise self‐assembly of the organic semiconductor materials with desired optoelectronic functions, typically the NIR emission with high PLQY and long emission wavelength.^[^ ^73^ ^]^ Inspired by these advantages, our group skillfully developed a unique cocrystal strategy to finely self‐assemble the organic CT cocrystal with NIR emission via the noncovalent interaction of CT interaction, which is different from the above four kinds of NIR materials via the rational molecular design and advanced synthetic technique.^[^ ^52^ ^]^ Moreover, the organic CT cocrystal of TP–F_4_TCNQ has been applied in NIR optical waveguides with a low optical loss coefficient (*R*) of 0.060 dB/μm at 770 nm.^[^ ^52^ ^]^ This prospective investigation provides a deeper insight into the development of organic NIR emitters for advanced organic optoelectronics or multichannel bioimaging.

## Conflict of Interest

The authors declare no conflict of interest.
